# Clinical characteristics of newly detected pharyngo-laryngeal lesions during transoral endoscopic surgery

**DOI:** 10.1007/s10147-026-03013-2

**Published:** 2026-03-18

**Authors:** Satoru Miyamaru, Daizo Murakami, Hidenori Katsura, Hideaki Miyamoto, Kenshi Matsuno, Sadahiro Yamamura, Narumi Hayashi, Toshinori Hirai, Yasuhito Tanaka, Yorihisa Orita

**Affiliations:** 1 Department of Otolaryngology-Head and Neck Surgery, Graduate School of Medicine, Kumamoto University, 1-1-1, Honjo, Chuo-ku, Kumamoto, 860-8556 Japan; 2 Department of Gastroenterology and Hepatology, Graduate School of Medicine, Kumamoto University, Kumamoto, Japan; 3 Department of Diagnostic Radiology, Graduate School of Medicine, Kumamoto University, Kumamoto, Japan

**Keywords:** Pharyngo-laryngeal carcinoma, Transoral endoscopic surgery, Newly detected lesion

## Abstract

**Background:**

Advances in image-enhanced endoscopy have improved early detection of superficial squamous cell carcinomas in the oropharynx, hypopharynx, and larynx. As a minimally invasive treatment, transoral endoscopic surgery offers advantages over (chemo)radiotherapy and open surgery, including preservation of voice and swallowing function and the option of future radiotherapy. However, some lesions remain undetected during preoperative examinations of conscious patients and are identified only intraoperatively under general anesthesia. In this study, we sought to clarify the clinical characteristics of lesions newly detected during surgery.

**Methods:**

We retrospectively reviewed 193 patients with 284 superficial squamous cell carcinoma lesions of the oro-hypopharynx or larynx who underwent transoral endoscopic surgery between January 2016 and December 2024. To identify factors associated with intraoperative detection, newly identified lesions were compared with preoperatively detected lesions in terms of location, tumor size, histopathology, and preoperative endoscopic examination conditions.

**Results:**

Among the 284 lesions, 24 (8.5%) were newly detected intraoperatively, which were significantly smaller than those detected preoperatively (median: 7.5 vs. 17 mm) and were mainly located on the posterior walls of the oropharynx and hypopharynx. Sixteen lesions were identified using narrow-band imaging (NBI) alone, whereas eight required additional Lugol staining. Lesions requiring Lugol staining were significantly more likely to be carcinoma in situ than invasive carcinoma.

**Conclusions:**

Intraoperatively detected lesions were generally smaller and more frequently located on the posterior pharyngeal wall. Given the limitations of conscious endoscopic examinations, meticulous intraoperative inspection using NBI and Lugol staining is essential to avoid overlooking indistinct lesions.

## Introduction

Recent advances in endoscopic imaging technologies, including narrow-band imaging (NBI) [1–4], together with the increasing tendency for gastrointestinal endoscopists to routinely observe the pharyngo-laryngeal region during examinations [5–8], have led to a more frequent early detection of oropharyngeal, hypopharyngeal, and laryngeal cancers. For superficial lesions detected at an early stage, transoral surgery (TOS), such as endoscopic laryngopharyngeal surgery (ELPS) and endoscopic submucosal dissection (ESD), has been reported to achieve favorable long-term outcomes [9–11] and has become widely adopted as a less invasive alternative to open surgery or (chemo)radiotherapy. In addition to its low invasiveness and less frequent occurrence of complications, TOS offers several notable advantages, including the preservation of vocal and swallowing functions and option to reserve radiotherapy for future treatment needs. Furthermore, even in patients who have undergone radiotherapy, early detection followed by TOS may contribute to an avoidance of highly invasive procedures that could result in significant functional impairment. At our institution, we have actively applied TOS for superficial pharyngeal and laryngeal cancers, the number of cases of which has risen in recent years.

Patients with squamous cell carcinoma (SCC) in the pharyngo-laryngeal region frequently develop multiple synchronous or metachronous lesions [9–11], and we have encountered cases in which lesions that were not identified preoperatively were newly detected during surgery. This may be attributable to the complex three-dimensional anatomy of the affected region, as well as physiological responses, such as the gag and cough reflexes, that limit prolonged and detailed observation during endoscopic examinations under conscious conditions. In contrast, during TOS under general anesthesia, the use of a rigid curved laryngoscope facilitates the wide exposure of the pharyngo-laryngeal area, thereby enabling meticulous inspection. Moreover, Lugol staining, which is conducive to the detection of superficial SCCs [12, 13] although difficult to employ under conscious conditions due to mucosal irritation, can also be combined with NBI to enable more detailed mucosal assessment. Consequently, lesions that are typically difficult to identify preoperatively may be more readily detected intraoperatively.

Acknowledging that certain lesions may evade detection during outpatient endoscopy, early identification and treatment of such lesions during TOS may contribute to an avoidance of (chemo)radiotherapy or function-sacrificing surgery. Accordingly, in this study, we sought to clarify the clinical characteristics of lesions newly identified during TOS under general anesthesia by comparing these with those detected preoperatively. We believe that the findings of this comparative study may provide important insights with respect to key considerations for thorough intraoperative inspection and the management of superficial pharyngo-laryngeal cancers.

## Materials and methods

In this study, we focused on patients who had undergone curative TOS for superficial oro-hypopharyngeal or laryngeal SCC at our institution between January 2016 and December 2024. The study was approved by the Ethics Committee of Kumamoto University (approval number: 2338), and written informed consent was obtained from all patients.

To determine the treatment strategy, in addition to flexible fiberoptic laryngoscopy and upper gastrointestinal endoscopy, we conducted physical examinations and imaging studies such as computed tomography and magnetic resonance imaging, on the cases subsequently reviewed by a multidisciplinary tumor board.

Flexible fiberoptic laryngoscopy was performed by otolaryngologists/head and neck surgeons using white-light imaging and NBI. To minimize the extent of blind areas during pharyngo-laryngeal observations, in all cases, we applied a modified version of Killian’s method [14, 15]. Upper gastrointestinal endoscopy was performed by experienced gastroenterologists or radiologists using high-definition magnifying endoscopes (primarily GIF-H260Z or GIF-H290Z; Olympus Medical Systems Corp., Tokyo, Japan). Examinations were conducted under sedation with pethidine hydrochloride, midazolam, or diazepam using white-light imaging and magnifying NBI. When appropriate, the Valsalva maneuver [16–18] was used to facilitate visualization of the hypopharynx.

During the informed consent process for surgery, patients were informed that pharyngo-laryngeal SCC may present with synchronous lesions at different sites. Accordingly, they were told that Lugol chromoendoscopy would be performed intraoperatively for detailed inspection of the pharyngo-laryngeal mucosa and that any newly detected lesions would be resected simultaneously. This information is explained verbally to the patients and is explicitly stated in the surgical consent form.

TOS was performed under general anesthesia. The endotracheal tube was fixed at the midline of the upper jaw, and a curved rigid laryngoscope (Nagashima Medical Instruments Co., Ltd., Tokyo, Japan) was used to elevate the larynx, thereby ensuring an adequate field of view and working space. The lesion boundaries were confirmed using magnifying NBI, followed by Lugol staining performed using a high-definition endoscope (primarily GIF-H260Z or GIF-H290Z; Olympus Medical Systems Corp., Tokyo, Japan). The margins of lesions were marked using a DualKnife J (KD-655Q; Olympus Medical Systems Corp., Tokyo, Japan). To elevate the mucosa, a solution consisting of a 1:1 mixture of isotonic sodium chloride solution and 0.4% sodium hyaluronate (MucoUp^®^, Seikagaku Corporation, Tokyo, Japan), with indigo carmine, was submucosally injected. Resection was performed using a DualKnife J and a VIO300D electrosurgical unit (Erbe Elektromedizin GmbH, Germany) in conjunction with single-channel endoscope (GIF-Q260J or GIF-H290T; Olympus Medical Systems Corp.) equipped with a transparent cap. During dissection, the tissue was retracted using forceps or ring-shaped threads [19].

Postoperatively, the patients underwent regular follow-up at an outpatient clinic, with examinations being conducted using a flexible fiberoptic laryngoscope. Upper gastrointestinal endoscopy was also performed once or twice a year. The frequency of clinical visits and endoscopic surveillance was adjusted based on pathological findings for the resected specimens. In patients in whom recurrence was suspected, a biopsy was performed for diagnosis, and the patient was reevaluated by the multidisciplinary tumor board to determine further treatment strategies.

To characterize lesions newly identified during TOS (intra-op lesions) and to identify factors associated with their detection, the following variables were analyzed. Clinical characteristics including tumor size, primary site (subsite), and pathological features (invasive carcinoma vs. carcinoma in situ) were compared between intra-op lesions and lesions identified preoperatively (pre-op lesions). Tumor size was evaluated as the maximum longitudinal diameter of the resected specimen. With respect to preoperative upper gastrointestinal endoscopy, the following factors were assessed: whether the lesion was retrospectively visible on recorded images, the purpose of the examination, the number of synchronous lesions, and the endoscopic system used. Visibility was determined retrospectively by reviewing recorded endoscopic images to assess whether the intra-op lesion could be identified. The purpose of the endoscopic examination was categorized as follows: (1) assessment of surgical indication for known pharyngo-laryngeal or esophageal cancer (i.e., cancer already diagnosed at another institution) or (2) post-treatment surveillance or screening in patients without known lesions. Because the presence of a known cancer or multiple synchronous lesions might divert attention from other areas, these variables were included in the analysis. Intra-op lesions located within the same subsite as a pre-op lesion were excluded from analysis, as such lesions were not considered clinically distinct entities.

Among intra-op lesions, clinical characteristics were also compared between lesions detectable by NBI alone and those requiring Lugol chromoendoscopy for identification. Furthermore, to evaluate the adequacy of treatment for intra-op lesions, resection margin status and recurrence rates were compared with those of pre-op lesions.

We also evaluated the presence of multiple synchronous and metachronous primary cancers, including the number of synchronous lesions, frequency of metachronous occurrences, and time to development of metachronous lesions.

Statistical analyses were performed using JMP Pro 14 software (SAS Institute Inc., Cary, NC, USA), with comparative analyses being conducted using the Wilcoxon rank-sum, Kruskal–Wallis, and Pearson’s chi-squared tests, with the level of significance set at p < 0.05.

## Results

In total, 193 patients with 284 lesions were included in this study (180 men, 13 women; median age: 70 years [range, 43–89 years]). The characteristics of these patients are summarized in Table 1. Of the 284 lesions, 212 were located in the hypopharynx, 49 in the oropharynx, and 23 in the larynx. The hypopharyngeal lesions were located in the pyriform sinus (n = 149), posterior wall (n = 43), or post-cricoid (n = 20), whereas those in the oropharynx were located on the posterior (n = 28), lateral (n = 11), and superior, or anterior (n = 5 each) walls. All laryngeal lesions were located in the supraglottic region.

**Table 1:** Characteristics of all patients

Number of Patients	193
Number of lesions	284
Gender	
Male	180
Female	13
Age, median [range] (years)	70 [43–89]
Primary site	
Hypopharynx	
Pyriform sinus	149
Posterior wall	43
Post-cricoid	20
Oropharynx	
Posterior wall	28
Lateral wall	11
Superior wall	5
Anterior wall	5
Larynx	
Supraglottic	23
T stages	
Tis	88
T1	140
T2	48
T3	8

Among these lesions, 24 were newly detected intraoperatively. All were amenable to curative resection during the same procedure. Detailed characteristics of these lesions are shown in Table 2. Among the 24 patients with these latter lesions, 21 were men and three were women (median age: 67 years [range, 50–83 years]). The posterior wall of the hypopharynx was identified as the most common lesional site (n = 13), followed by the pyriform sinus (n = 4), post-cricoid region (n = 3), posterior wall of the oropharynx (n = 3), and supraglottis (n = 1), and we noted a significant difference in the subsite distribution of the pre- and intra-op lesions, particularly for those located in the hypopharynx (p < 0.01; Table 3). Although owing to the small number of oropharyngeal lesions, statistical analysis was not performed, all these lesions were located on the posterior wall.

**Table 2:** Characteristics of newly detected lesions during transoral surgery

Number of lesions	24
Gender	
Male	21
Female	3
Age, median [range] (years)	67 [50–83]
Primary site	
Hypopharynx	
Pyriform sinus	4
Posterior wall	13
Post-cricoid	3
Oropharynx	
Posterior wall	3
Larynx	
Supraglottic	1
T stages	
Tis	13
T1	10
T2	1
Detection method	
NBI	16
NBI+Lugol	8

*NBI* narrow-band imaging

**Table 3:** Comparison of subsites between pre- and intraoperative lesions

Local	Subsite	Pre-op	Intra-op	P value
Hypopharynx	Pyriform sinus	145	4	<0.001
	Posterior wall	30	13	
	Post-cricoid	17	3	
Oropharynx	Posterior wall	25	3	0.494
	Lateral wall	11	0	
	Superior wall	5	0	
	Anterior wall	5	0	
Larynx	Supraglottic	22	1	−
Total		260	24	

*Pre-op* lesions identified preoperatively, *Intra-op* lesions newly identified during transoral surgery

Whereas pre-op lesions ranged in size from 1 to 58 mm (median: 17 mm), intra-op lesions were found to be significantly smaller, ranging in size from 2 to 24 mm (median: 7.5 mm) (p < 0.01). Details of the sizes of tumors according to location are presented in Table 4. Even when stratified by subsite (hypopharyngeal posterior wall, pyriform sinus, and post-cricoid), intra-op lesions were significantly smaller than pre-op lesions (p < 0.001, p = 0.004, and 0.017, respectively). Pathologically, among pre-op lesions, 185 were invasive carcinoma and 75 were carcinoma in situ. In contrast, among intra-op lesions, 11 were invasive carcinoma and 13 were carcinoma in situ. The proportion of carcinoma in situ was significantly higher in intra-op lesions (p = 0.010).

**Table 4:** Comparison of pre- and intraoperative lesion tumor size

Local	Subsite	Pre-op		Intra-op		P value
		n	Median [mm]	n	Median [mm]	
Hypopharynx	Pyriform sinus	145	16 (2–58)	4	6 (3–10)	0.004
	Posterior wall	30	18.5 (7–42)	13	8 (2–24)	<0.001
	Post-cricoid	17	18 (5–41)	3	6.5 (2–9)	0.017
Oropharynx	Posterior wall	25	13.5 (1–43)	3	13 (6–19)	0.832
Larynx	Supraglottic	22	14 (2–47)	1	10	−
Total		260	17 (1–58)	24	7.5 (2–24)	<0.001

Pre-op: lesions identified preoperatively, *Intra-op* lesions newly identified during transoral surgery

Clinical details of all 24 intra-op lesions are shown in Table 5. Regarding tumor size, lesions smaller than the median size of intra-op lesions (7.5 mm) were denoted as “++,” lesions larger than the median size of pre-op lesions (17 mm) as “–,” and those between 7.5 and 17 mm as “+.” The purpose of preoperative endoscopy was categorized as follows: “I” for examinations performed to assess indications for endoscopic surgery in patients with already diagnosed pharyngo-laryngeal or esophageal cancer and “S” for post-treatment surveillance or screening in patients without known lesions. With respect to the endoscopic system, newer high-resolution systems were categorized as “Later,” and older lower-resolution systems as “Earlier.” Retrospective review of recorded images showed that only 7 of the 24 intra-op lesions were retrospectively visible, whereas most lesions had not been recognized at the time of examination. The visible lesions were located in the hypopharyngeal posterior wall (n = 3), oropharyngeal posterior wall (n = 2), pyriform sinus (n = 1), and supraglottic region (n = 1), with no apparent subsite tendency. These lesions were considered to have been overlooked because of their small size and/or pathological diagnosis as carcinoma in situ. When comparing intra-op and pre-op lesions with respect to preoperative endoscopic factors, no significant differences were observed in the number of synchronous lesions, purpose of examination, or endoscopic system used (p = 0.847, 0.904, and 0.218, respectively).

**Table 5.** Factors associated with lesion detection

Lesion number	Visibility	Location	Size	Pathology	Number of lesions	Purpose of endoscopic examination	Endoscopy system
1	Yes	Posterior wall (O)	++	CIS	2	I	Later
2*	Yes	Pyriform sinus	++	CIS	3	I	Later
3	Yes	Posterior wall (H)	++	SCC	2	I	Earlier
4	Yes	Posterior wall (H)	+	SCC	2	S	Later
5	Yes	Supraglottic	+	SCC	2	S	Later
6†	Yes	Posterior wall (O)	–	CIS	3	S	Later
7†	Yes	Posterior wall (H)	–	SCC	3	S	Later
8	No	Post-cricoid	++	CIS	2	S	Earlier
9	No	Posterior wall (H)	++	CIS	2	S	Later
10*	No	Posterior wall (H)	++	CIS	3	I	Later
11	No	Posterior wall (H)	++	CIS	3	I	Later
12	No	Pyriform sinus	++	CIS	2	I	Later
13	No	Pyriform sinus	++	CIS	2	I	Later
14	No	Post-cricoid	++	SCC	2	S	Later
15	No	Posterior wall (H)	++	SCC	3	I	Later
16	No	Posterior wall (H)	++	SCC	4	I	Later
17	No	Post-cricoid	+	CIS	2	I	Later
18	No	Posterior wall (H)	+	CIS	2	S	Later
19	No	Posterior wall (O)	+	CIS	3	I	Later
20	No	Posterior wall (H)	+	SCC	2	S	Later
21	No	Posterior wall (H)	+	SCC	2	I	Later
22	No	Pyriform sinus	+	SCC	2	S	Later
23	No	Posterior wall (H)	–	CIS	2	I	Later
24	No	Posterior wall (H)	–	SCC	2	I	Later

*CIS* carcinoma in situ, *SCC* squamous cell carcinoma, *NBI* narrow-band imaging, *I* evaluation for surgical indication, *S* post-treatment surveillance, *Later* later-generation system, *Earlier* earlier-generation system

^*^and ^†^ indicate the same patients, (*H*) hypopharyngeal lesion; (*O*) oropharyngeal lesion; ++: <7.5 mm, +: 7.5–17 mm, -: >17 mm

Whereas 16 of the newly identified lesions were detected using NBI alone, eight were identified with supplementary use of Lugol staining, the details of which are presented in Table 6. Although we detected no significant differences between the NBI-only and NBI-plus-Lugol groups with respect to subsite distribution (p = 0.539), in terms of size, those detected using only NBI (between 2 and 24 mm; median: 8.5 mm) tended to be larger than those identified using NBI-plus-Lugol (between 2 and 19 mm; median, 6 mm); however, the difference was not statistically significant (p = 0.231). Pathologically, among the 16 lesions detected by NBI alone, 10 were invasive carcinoma and 6 were carcinoma in situ. In contrast, among the 8 lesions detected using additional Lugol staining, 1 was invasive carcinoma and 7 were carcinoma in situ. Lesions detected with the aid of Lugol staining were therefore significantly more likely to be carcinoma in situ (p = 0.034).

**Table 6:** Characteristics of lesions according to detection method

	NBI	NBI + Lugol
Number of cases	16	8
Median tumor size [mm]	8.5 (2–24)	6 (2–19)
Pathological diagnosis		
Carcinoma in situ	7	6
Microinvasive carcinoma	9	2
Tumor location		
Hypopharynx		
Pyriform sinus	2	2
Posterior wall	10	3
Post-cricoid	2	1
Oropharynx		
Posterior wall	1	2
Larynx		
Supraglottic	1	0

*NBI* narrow-band imaging

Among the 24 patients with newly detected lesions, the lesions in two patients had positive horizontal margins with carcinoma *in situ*, and one of these patients developed a local recurrence and underwent a second operation using TOS. However, there were no significant differences between the intra- and pre-op groups with respect to the surgical margins and oncological outcomes, including local recurrence, indicating that the intra-op lesions were safely and appropriately resected (Table 7).

**Table 7:** Proportion of positive surgical margins and local recurrence

	Positive margin		Local recurrence	
	Pre-op	Intra-op	Pre-op	Intra-op
Hypopharynx				
Pyriform sinus	19/145	0/4	1/145	0/4
Posterior wall	3/30	1/13	1/30	1/13
Post-cricoid	1/17	0/3	0/17	0/3
Oropharynx				
Posterior wall	0/25	1/3	2/25	0/3
Larynx				
Supraglottic	6/22	0/1	2/22	0/1
Total	29/239	2/24	6/239	1/24

*Pre-op* lesions identified preoperatively, *Intra-op* lesions newly identified during transoral surgery

Fifty-one patients underwent resection of multiple lesions in a single operation, accounting for 22.8% of the 224 surgical cases. Specifically, 41 patients had two lesions, nine had three, and one had four. In addition, during the follow-up period, 23 patients (10.3%) underwent multiple operations for metachronous lesions, among whom, 17 patients underwent two operations, five underwent three, and one underwent four. Among the 30 total metachronous events, the time interval from the previous surgery to the detection of a new lesion ranged from 5 to 54 months (median: 14.5 months).

## Discussion

The pharyngeal region, particularly the oro- and hypopharynx, is highly susceptible to carcinogenic factors such as smoking and alcohol consumption, which can contribute to inducing widespread genetic mutations and epigenetic modifications throughout the mucosa, leading to an accumulation of precancerous lesions and development of multiple independent cancers, either synchronously or metachronously. This phenomenon, referred to as field cancerization, was first described by Slaughter et al. in 1953 [20]. In the present study, we observed multiple synchronous lesions in 51 of 224 patients (22.8%), in 24 of whom, these lesions were newly detected intraoperatively despite not being identified during preoperative endoscopy.

Newly identified intraoperative lesions were most frequently located on the posterior wall of the hypopharynx and were significantly smaller than the preoperatively detected lesions, both overall and among the different subsites. Further, the proportion of carcinoma in situ was significantly higher among intra-op lesions than among pre-op lesions. In their analysis of 58 patients undergoing TOS for superficial oro-hypopharyngeal cancer, Ono et al. reported that the mean number of detected lesions per patient increased from 1.17 during preoperative endoscopy to 1.47 during endoscopy under general anesthesia [21], thus indicating that synchronous lesions undetected in conscious conditions, are often identified intraoperatively. These newly detected lesions tend to be small and lack prominent color or surface irregularities, with no discernable subsite-specific differences with respect to detectability.

Similar findings have been reported in other studies. For example, in a prospective evaluation of intraoperative surveillance using NBI combined with Lugol staining, Kimura et al. identified 13 previously undetected lesions (SCC or severe dysplasia) in 10 of 78 patients, with the pyriform sinus being the most frequently lesioned site [22]. Furthermore, on the basis of a review of endoscopic images from routine upper gastrointestinal tract examinations, Nakajo et al. defined “missed cancers” as pharyngo-laryngeal cancers that were not detected within 15 months prior to diagnosis [23]. Among 240 lesions, 85 (35.4%) met this definition, with these lesions typically being less than 13 mm in size and frequently located on the lingual surface of the epiglottis/vallecula or the pyriform sinus.

The findings of previous studies have thus indicated that newly detected or missed lesions often arise in the pyriform sinus [22, 23], epiglottis, and vallecula [23]. Moreover, whereas it has previously been established that conventional endoscopic imaging has lower diagnostic accuracy in subsites such as the pyriform sinus and post-cricoid region [24], in the present study, intraoperatively detected lesions were found to be less frequently detected in the pyriform sinus and vallecula, being predominantly observed on the posterior walls of the oropharynx and hypopharynx. We suspect that this discrepancy may reflect our routine use of a modified Killian method during flexible laryngoscopy and the Valsalva maneuver with a mouthpiece during upper gastrointestinal endoscopy, both of which contribute to enhancing the visibility of the pyriform sinus. However, visualization of the hypopharyngeal posterior wall remains limited during conscious endoscopy owing to obstruction from the laryngeal framework (thyroid, cricoid, and arytenoid cartilages), as well as contraction of the upper esophageal sphincter. Lesions located in the vicinity of the midline or immediately above the esophageal inlet do not expand sufficiently even when using the aforementioned techniques, and thus remain blind spots. Furthermore, during flexible fiberoptic laryngoscopy, the posterior wall is observed in a tangential direction, making it difficult to evaluate and potentially prone to being overlooked. Consequently, detailed evaluations of the posterior wall are generally only feasible having initially established sufficient exposure, such as that obtained using a curved laryngoscope under general anesthesia.

Among the 13 newly detected posterior hypopharyngeal wall lesions in the present series, 10 were identified using NBI alone. In this regard, the findings of previous studies have revealed that hypopharyngeal posterior wall cancers tend to be diagnosed at more advanced stages than tumors in other subsites [25, 26], thus highlighting the inherent difficulty of early detection in this region. Surgeons and endoscopists should accordingly be aware of the limitations of standard preoperative endoscopy in detecting posterior wall lesions and remain particularly vigilant when planning TOS.

Despite careful inspection based on NBI during laryngeal exposure, eight lesions in the cohort described herein were identified only after performing Lugol staining. These lesions showed a significantly higher proportion of carcinoma in situ compared with those detected by NBI alone. Tsuji et al. have reported that lesions newly identified intraoperatively on the posterior wall of the oropharynx exhibited less neovascularization than lesions detected preoperatively, which may explain their invisibility on NBI [27]. Immunohistochemical evaluation using CD34, a vascular marker, demonstrated significantly lower microvascular density in newly detected lesions compared with known lesions. Because NBI enhances visualization of the epithelial microvascular architecture, lesions with limited microvascular proliferation may be difficult to detect using NBI alone. These findings are consistent with those reported by Ono et al., who noted that newly detected lesions often lack distinctive changes in color or surface irregularities [21]. Lesions with relatively preserved intrapapillary capillary loop (IPCL) architecture have generally been associated with carcinoma in situ or superficially invasive disease [28], which is consistent with our finding that lesions detected only with the aid of Lugol staining were more frequently carcinoma in situ. These lesions were therefore likely difficult to detect using NBI alone. Furthermore, in superficial esophageal SCC, lesions that are undetectable when using NBI alone, but detectable with Lugol staining, are often associated with multiple background Lugol-voiding areas [29]. Chronic inflammation, such as that caused by smoking and alcohol consumption, may generate numerous irregular Lugol-voiding areas, thereby hampering the detection of lesions using NBI, owing to expanded microvascular patterns. Given that the pharynx is even more susceptible to chronic inflammation than the esophagus [22], Lugol staining may make a particularly important contribution to identifying subtle lesions. Kimura et al. [22] also demonstrated that although eight of 13 newly detected lesions were identifiable using NBI alone, all were confirmed with Lugol staining, thereby highlighting its complementary value. However, despite this utility, it has been established that Lugol solution causes intense mucosal irritation, with associated risks such as pain, gagging, vomiting, and aspiration-related lung injury [30], and, consequently, its application is generally avoided when examining conscious patients. Under general anesthesia, however, its use is considered essential for a comprehensive mucosal evaluation.

The incidence of metachronous lesions in patients with superficial oro-hypopharyngeal SCC has been reported to range from 16% to 26.6% [10, 31–33], which compares with the 11.9% (23 of 193 patients) incidence detected in the present study. Given the relatively short follow-up period for many patients, it is reasonable to assume that the frequency of metachronous cancers detection would be higher with longer observation periods. Accordingly, postoperative surveillance should focus not only on recurrence but also on the early detection of multiple metachronous cancers. Therefore, long-term follow-up for as long as possible, preferably lifelong, is desirable. When long-term follow-up at a single institution is difficult, referral to other institutions or encouraging patients to undergo regular health checkups independently should be considered. Appropriate patient education is essential to facilitate this process.

Despite our important findings, this study does have certain limitations. Notably, this was a retrospective single-institution study with a relatively small sample size. Moreover, in some cases, the interval between preoperative endoscopy and surgery was prolonged, and thus new lesions may have developed during the intervening periods. In addition, given that the follow-up duration was limited, long-term prognostic outcomes and safety assessments were not fully evaluated. Consequently, a multicenter, prospective, long-term study with a larger cohort is warranted to validate our findings.

## Conclusion

In this study, we examined the clinical characteristics of lesions that although went undetected during preoperative endoscopic evaluation, were newly detected during TOS under general anesthesia. These lesions were significantly more likely to be located on the posterior wall of the oropharynx or hypopharynx and were generally smaller than those detected preoperatively. TOS for superficial pharyngo-laryngeal cancers is highly valuable in that it enables preservation of the vocal and swallowing functions of patients and retains future options for (chemo)radiotherapy, making early detection particularly important. Given the high incidence of multiple synchronous and metachronous cancers in this region and the inherent limitations of preoperative endoscopic detection, careful intraoperative assessment to identify additional lesions is essential for optimal management.

## Data Availability

The datasets generated and analyzed in the current study are available from the corresponding author upon reasonable request.
